# circRNA_SLC8A1 promotes the survival of mycobacterium tuberculosis in macrophages by upregulating expression of autophagy-related protein SQSTM1/p62 to activate the NF-κB pathway

**DOI:** 10.1038/s41598-024-55493-9

**Published:** 2024-03-04

**Authors:** Zhenyun Li, Yuan Gao, Bianfang Zhang, Wei Dong, Yuling Xi, Yan Li, Junwei Cui

**Affiliations:** 1 Department of Tuberculosis, The First Affiliated Hospital of Xinxiang Medical University, Weihui, 453100 Henan China; 2 Clinical Pharmacy Office, The First Affiliated Hospital of Xinxiang Medical University, Weihui, 453100 Henan China; 3 Gastrointestinal Surgery, The First Affiliated Hospital of Xinxiang Medical University, Weihui, 453100 Henan China

**Keywords:** Mycobacterium tuberculosis, Macrophages, circRNA_SLC8A1, miR‐20b‐5p, SQSTM1/p62, The NF-κB signaling pathway, Cell biology, Immunology, Molecular biology, Medical research

## Abstract

Macrophages act as the first immune defense line of the host against Mycobacterium tuberculosis (Mtb). A previous study showed that circRNA_SLC8A1 was significantly upregulated in Mtb-infected macrophages, but its regulatory mechanism in anti-tuberculosis infection is unclear. Therefore, this study aimed to investigate the role of circRNA_SLC8A1 in the anti-tuberculosis activity of macrophages. We showed that circRNA_SLC8A1 was upregulated in tuberculosis patients. Moreover, the binding sites of miR-20b-5p on circRNA_SLC8A1 and Sequestosome 1 (SQSTM1/p62) mRNA were predicted by StarBase and verified by the double luciferase reporter gene assay. Next, we found that miR-20b-5p expression was decreased, while SQSTM1 protein expression was increased in a time- and dose-dependent manner in the human macrophage U937 in response to Mtb infection. Furthermore, circRNA_SLC8A1 overexpression vector (circRNA_SLC8A1) or shRNA (sh-circRNA_SLC8A1) and/or miR-20b-5p mimic or inhibitor and/or SQSTM1 overexpression vector (SQSTM1) or small interfering RNA (si-SQSTM1) or its corresponding control were transfected into Mtb-infected macrophages. Results showed that overexpression of circRNA_SLC8A1 or miR-20b-5p inhibitor promoted the secretion of pro-inflammatory factors IL-1β, IL-6, and TNF-α, increased Nitric Oxide (NO) content and inducible nitric oxide synthase (iNOS) expression, inhibited Reactive oxygen species (ROS) production. Cleaved-caspase-3 protein expression, and cell apoptosis, and promoted Mtb survival. Silencing SQSTM1 inhibited secretion of pro-inflammatory factors and activation of the NF-κB pathway. Overexpression of miR‐20b‐5p blocked the promoting of circ‐SLC8A1 on SQSTM1 protein expression. In summary, circRNA_SLC8A1 sponged miR‐20b‐5p to upregulate SQSTM1/p62 expression and promoted Mtb survival in macrophages through the NF-κB signaling pathway.

## Introduction

Tuberculosis is an infectious disease with a high mortality rate, making for more than 10 million people becoming newly sick and 1.6 million deaths each year. Mycobacterium tuberculosis (Mtb) is a bacterium that develops pulmonary tuberculosis when it infects the lungs^1^. According to incomplete statistics, about a quarter of the world's population is infected with Mtb^2^. Although the incidence of Mtb infection in humans is high, especially in developing countries with large populations, most infected people in these infected populations are asymptomatic (known as latent tuberculosis), and only a relatively small proportion (5%-10%) of infected people develop active tuberculosis infection during their lifetime^3,4^. Bacillus Calmette Guérin (BCG) is currently used in infants to prevent Mtb infection, however, its effect is not satisfactory for adults. So far, there is no effective vaccine to prevent all Mtb strains^5^. At the same time, current drug treatments face drug resistance barriers and current methods of diagnosing tuberculosis, are inefficient^6,7^. With all this in mind, there is an urgent need to elucidate the pathological mechanisms of Mtb to identify effective therapeutic targets and diagnostic biomarkers.

Macrophages are the main host immune response cells of the body and affect the intracellular growth and persistence of Mtb during all phases of tuberculosis, from primary infection with bacillary dissemination, through latency and reactivation tuberculosis^8,9^. Activated macrophages could clear intracellular infected Mtb. In contrast, Mtb could survive and disseminate by inducing death of infected macrophages^9,10^. Bacterial dissemination is enhanced after death of macrophages infected with Mtb. This may be due to extracellular growth of tissues releasing Mtb or less clearance of Mtb by dead macrophages^11,12^. Meanwhile, Mtb also activates various mechanisms to weaken the immune response of macrophages and create an environment conducive to their survival^13^. A range of defense mechanisms, including autophagy, cell apoptosis, and inflammatory responses, are essential for the control of Mtb infection^11^. Therefore, inhibition of the inflammatory response and apoptosis of macrophages following Mtb infection is critical for controlling Mtb infection.

Macrophage heterogeneity and plasticity are tightly regulated at multiple transcriptional levels^14^. Among them, it has been reported that Circular RNAs (circRNAs) plays an important role in processes like host defense against Mtb infection and survival of Mtb within the host^15^. circRNAs are a special type of noncoding RNAs that form a closed loop by covalent bonds between the 3’ and 5’ ends. Recent evidence suggested that circRNAs have been recognized as regulatory molecules involved in the development and progression of a range of human diseases, including tuberculosis. In tuberculosis, circRNAs have been shown to modulate host anti-tuberculosis immune responses, such as reducing cell apoptosis and regulating autophagy^15–17^. A previous study identified 142 circRNAs that were aberrantly expressed in tuberculosis patients, 32 upregulated and 110 downregulated, which implied a potential role of circRNAs for diagnosing tuberculosis. Among them, circRNA_SLC8A1 was found to be significantly upregulated in Mtb infected macrophages^18^, but its specific regulatory mechanism in tuberculosis is unclear. Therefore, the present study investigated the role of circRNA_SLC8A1 in tuberculosis and attempted to find effective diagnostic biomarkers for tuberculosis, providing a new strategy for tuberculosis diagnosis and treatment.

## Materials and methods

### Patients sample collection

This study prospectively included 126 tuberculosis patients (TB) diagnosed in the First Affiliated Hospital of Xinxiang Medical University from March 2018 to October 2022. According to the condition, the patients were assigned into three subgroups, including 51 cases of active pulmonary tuberculosis (PTB, median age 48.6 ± 16.3 years; male 47.1%), 33 cases of latent pulmonary tuberculosis infection (LPTB, median age 45.7 ± 10.5 years; male 48.5%), and 42 cases of extra-pulmonary tuberculosis infection (EPTB, median age 47.9 ± 15.1 years; male 54.8%). In addition, 49 healthy volunteers (HC, median age 49.0 ± 12.4 years; male 46.9%) who accepted physical examination in our hospital during the same period were selected. All PTB patients were diagnosed with tuberculosis based on the typical clinical symptoms of tuberculosis, bacterial culture, and imaging studies^19,20^. And patients younger than 18 years old, with viral hepatitis B, liver cirrhosis, HIV positive, pregnant or tumor status, and long-term use of immunosuppressants such as hormones were excluded. At the time of blood sample collection, 8 of 51 PTB patients have received the first-line anti-Mtb infection treatment (drug regimen: rifampicin, isoniazid, ethambutol, or pyrazinamide) for less than 3 days. The clinical characteristics of all subjects are described in Table 1. On the day of outpatient or admission, 5 mL of whole blood samples were collected from all subjects, centrifuged at 1500×*g* at 4 °C for 10 min, and stored at − 80 °C. All patients provided informed consent, and the protocol conformed to the guidelines of the Declaration of Helsinki. This study was approved by the ethics committee of the First Affiliated Hospital of Xinxiang Medical University and informed consents were obtained from all participants.

**Table 1:** The clinical characteristics of all subjects.

Variables	HC (n = 49)	Tuberculosis patients (TB, n = 126)			F/χ^2^	*P*-value
		PTB (n = 51)	LPTB (n = 33)	EPTB (n = 42)		
Age (years)	49.0 ± 12.4	48.6 ± 16.3	45.7 ± 10.5	47.9 ± 15.1	0.139	2.194
Gender					4.543	0.208
Male	23 (46.9%)	24 (47.1%)	16 (48.5%)	23 (54.8%)		
Female	26 (53.1%)	27 (52.9%)	17 (51.5%)	19 (45.2%)		
Bandim TBscore						
< 8	–	32 (62.7%)	–	–		
≥ 8	–	19 (37.3%)	–	–		
Teament						
Anti-TB	–	8 (15.7%)	–	–		
Other	–	0	–	–		
Treatment time						
< 3 days	–	8 (15.7%)	–	–		
≥ 3 day	–	0	–	–		

### Tuberculosis-associated severity indices

The modified Bandim TBscore was used to evaluate the severity in patients with PTB at the time of diagnosis, before the initiation of anti-Tuberculosis treatment^21,22^. It considers five symptoms (cough, hemoptysis, dyspnea, chest pain, and night sweats) and seven clinical findings (anemia, pulse > 90 beats/min, positive finding at lung auscultation, temperature > 37 °C (axillary), body mass index (BMI) < 18 and < 16, hypoproteinemia, and bilateral lung involvement in CT or X-ray). Each variable contributes one point. BMI contribute an extra point, if BMI < 16; hence the maximum score is 13 (Table 2). A score of 8 or greater is defined as moderate/severe TB, and patients were stratified into two severity classes: mild (Bandim TBscore < 8) and moderate/severe (Bandim TBscore ≥ 8).

**Table 2:** The modified bandim TBscore.

Variables	Modified TB score
Symptoms	
Cough	1
Hemoptysis	1
Dyspnea	1
Chest pain	1
Night sweats	1
Clinical Signs	
Anemia	1
Pulse > 90 beats/min	1
Positive finding at lung auscultation	1
Temperature > 37 °C	1
Body mass index (BMI) < 18	1
Body mass index (BMI) < 16	1
Hypoproteinemia	1
Bilateral lung involvement	1
Total	13

### Cells, bacterial culture, and infection

Human macrophages (U937 cells) and HEK-293 T cells and Mycobacterium tuberculosis H37Ra and H37Rv strains were purchased from ATCC company. HEK-293 T cells were maintained in DMEM media containing 10% fetal bovine serum (FBS; Thermo Fisher), the human macrophages were cultured in RPMI 1640 medium (Sigma, St. Louis, MO) containing 10% FBS, 4 mM L-glutamine, 100 U/mL penicillin and 100 mg/mL streptomycin, 10 mM HEPES and 1 mM sodium pyruvate (Sigma). All cells were incubated at 37℃ and 5% CO_2_. H37Rv and H37Ra strains were grown in Middlebrook 7H9 broth medium or 7H9 liquid medium supplemented with oleic acid albumin dextrose catalase enrichment (Becton and Dickinson, Cockeysville, MD) at 37℃. H37Ra and H37Rv were allowed to infect U937 cells at a multiple infection (MOI) of 1, 5 or 10, or at a MOI of 5 for 12, 24 and 48 h. Subsequently, cells were gently washed three times with phosphate-buffered saline (PBS) and then incubated with a complete medium containing 50 μg/mL gentamicin (Sigma-Aldrich) for 1 h at 37 °C to remove extracellular bacteria.

### pcD2.1- circRNA_SLC8A1 vector and pcDNA3.1 SQSTM1/p62 vector construction

Human circRNA_SLC8A1 sequence was cloned into the pcD2.1-circRNA_vector (Geneseed Biotech, Guangzhou, China). To knockdown circRNA_SLC8A1, shRNA-circRNA_SLC8A1 was synthesized and inserted into pcD2.1-circRNA_vector. Human SQSTM1/p62 CDS sequence was cloned into the pcDNA3.1 vector (Thermo Fisher Scientific, Shanghai, China).

### Transient transfections

Cells were seeded into 6-well plates at a density of 3 × 10^5^ cells/well in antibiotic-free medium and confluent to 80%. miR-20b-5p mimics and inhibitors, pcDNA3.1-SQSTM1/p62 overexpression vector, pcD2.1-circRNA_SLC8A1 plasmids and pcD2.1-sh-RNA circRNA_SLC8A1 plasmids or their corresponding negative controls were transfected into U937 cells by using Lipofectamine 2000 reagent (Invitrogen, Carlsbad, CA), according to the manufacturer's protocol.

### Luciferase Reporter Gene Assay

The relationship between miR-7-5p and RHPN1-AS1 or miR-7-5p and EGFR were verified by the dual- luciferase reporter gene assay. In brief, the pmirGLO vectors (Promega, Madison, WI, USA) containing the sequences of circRNA_SLC8A1 wild type or mutant with the mutated miR-20b-5p binding sites, or containing the sequences of wild type or mutant of SQSTM1/p62 3′-UTR with the miR-20b-5p binding sites were inserted into the pmirGLO vectors (Promega, Madison, WI, USA) for the construction of reporters. The HEK-293 T cells were co-transfected with above the pmirGLO vectors and miR-20b-5p mimic or NC mimic. After 48 h, the luciferase activity was determined with the Luciferase Reporter Gene Assay Kit (Promega).

### Colony-forming unit (CFU) assay

To assay the bacterial viability within human macrophages, U937 cells were infected with 5 MOI Mtb for 48 h at 37 °C under a 5% CO_2_ atmosphere. Subsequently, cells were washed three times with PBS to remove extracellular bacteria and then incubated with a complete medium containing 50 μg/mL gentamicin (Sigma-Aldrich) for 1 h at 37 °C. The infected cells were lysed with 0.5% Triton X-100 at 37 °C for 30 s, serially diluted with tenfold serial dilutions, coated with Mycobacterium solid culture medium (Gene-Optimal, China), and cultured at 37 °C for 3 weeks; then, CFUs were calculated using standard procedures.

### Cell counting kit 8 (CCK-8) assay

The activity of U937 cells was assay by Cell Counting Kit-8 (CCK-8) kit. U937 cells at a density of 5 × 10^3^ cells/well were seeded into 96-well plates, and cultured in a humidified atmosphere of 5% CO_2_ at 37 °C. Following 0, 24, 48, 72 h incubation, 10 μL of CCK-8 solution was respectively added into a 96-well plate and incubated for 2 h at 37℃. The wavelength of the microplate reader (Bio-Rad, Hercules, CA, USA) was used to determine the optical density (OD) value at 450 nm.

### Apoptosis assay

Annexin V-FITC/PI Apoptosis Detection Kit (vazyme, Nanjing, China) was used for detecting cell apoptosis. In brief, U937 cells were trypsinized, centrifuged, washed, and re‐suspended in 100 μL of binding buffer with a density of 5 × 10^5^ cells/mL, followed by adding 5 μL of Annexin V‐FITC and 5 μL of propidium iodide (PI) and incubated in the dark for 10 min. The cell apoptosis ratio was analyzed by flow cytometry (BD Biosciences).

### Enzyme-linked immunosorbent assay (ELISA)

The levels of interleukin-1β (IL-1β), interleukin-6 (IL-6) and tumor necrosis factor-α (TNF-α) were detected with ELISA kits (Dakewei, Shanghai), according to the manufacturer's protocols.

### Griess assay

U937 cell supernatants were collected for assessing the production of nitrite using Griess reagent (Promega), according to the manufacturer s instructions. The microplate reader was used for detecting absorbance of samples at 540 nm; nitrite concentrations were calculated using a standard curve.

### Measurement of intracellular ROS

Intracellular ROS levels were measured with ROS production was evaluated by ROS-sensitive fluorescent probe 5-(and-6)-chloromethyl-2',7'-dichlorodihydrofluorescein, acetyl ester (CM-H2DCFDA) (Invitrogen, Life Technologies, Ltd.). Briefly, macrophages at the density of 2 × 10^5^ cells/well were incubated with CM-H2DCFDA (10 μM/well) for 30 min at room temperature in the dark, and ROS production was detected by measuring the increase in fluorescence recorded at 495 nm excitation and 527 nm emission, by using a microplate reader (Varian Cary Eclipse Fluorescence Spectrophotometer.

### Western blotting

After infection for 12 h, U937 cells were collected and lysed with lysis buffer, and the protein concentration was measured with a Bradford protein assay kit. Thirty micrograms of total protein were separated by 10% SDS-PAGE and transferred to PVDF membranes. Membranes were blocked by 5% BSA for 1 h at room temperature, and incubated with primary antibodies, including Rabbit monoclonal anti-SQSTM1/p62 (1:1000, ab109012, Abcam), Rabbit monoclonal anti-Caspase-3 antibody (1:1000, ab32351, Abcam), Rabbit monoclonal anti-Cleaved Caspase-3 antibody (1:1000, ab32042, Abcam), Mouse monoclonal anti- Lamin B antibody (1:1000, ab232731, Abcam), Mouse monoclonal antibody anti-β-actin (1:1000, ab8226, Abcam) and human polyclonal antibody anti-GAPDH (1:1000, ab8245, Abcam) at 4 °C overnight. Then the membranes were incubated with specific secondary antibody goat Anti-mouse IgG (1:1000, ab150113, Abcam) for 2 h at room temperature. All immunoblots were visualized with chemiluminescence reagents (Millipore, Billerica, MA, USA). The protein levels were quantitated by a ChemiDoc XRS Imaging System (Bio-Rad Laboratories, Hercules, CA).

### Immunofluorescence analysis

U937 cells were fixed with 4% paraformaldehyde for 15 min and then permeabilized with Triton X-100 (0.1%), and subsequently blocked with 5% goat serum for 2 h at room temperature. Next, primary antibody anti-SQSTM1/p62 (1:200, ab109012, Abcam), anti-p-NF-κB (p-p65) (1:1000, ab278777, Abcam), was added and cultivated overnight at 4 °C. Alexa fluor fluorescein conjugated secondary antibody (1:500, ab150077, Abcam) was replenished and incubated at room temperature for 1 h. Finally, DAPI was incubated at room temperature for 10 min. Fluorescence intensity was evaluated by fluorescence microscopy (MF43-N, Mshot, China).

### RNA isolation and quantitative RT-PCR (RT‑qPCR)

**Figure 1:**
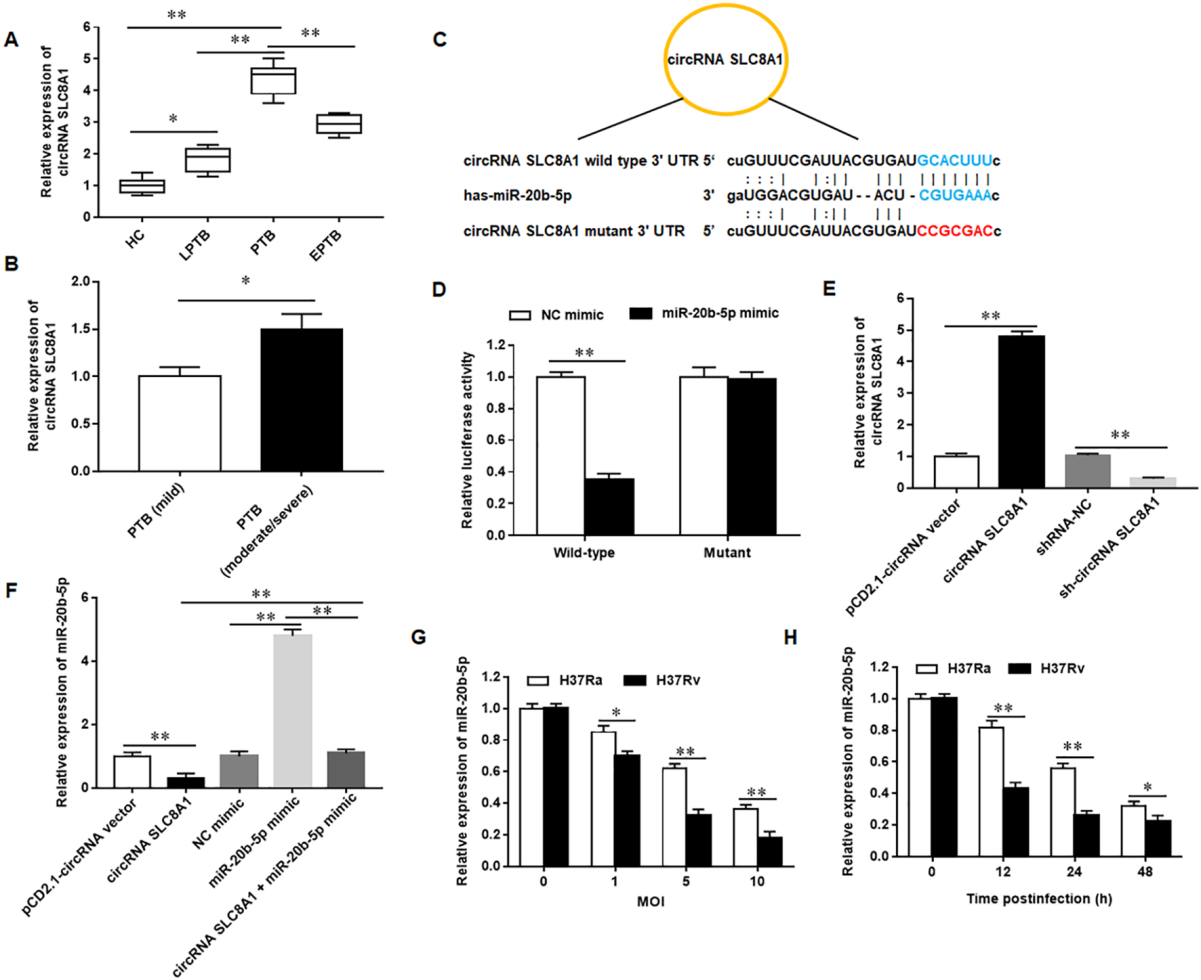
circRNA_SLC8A1 as a sponge for miR-20b-5p is upregulated in tuberculosis patients (**A**). The expression of circRNA_SLC8A1 in the peripheral blood of active pulmonary tuberculosis (PTB), latent pulmonary tuberculosis infection (LPTB), extra-pulmonary tuberculosis infection (EPTB) patients, and healthy volunteers (HC) (**B**). The expression of circRNA_SLC8A1 in the peripheral blood of PTB patients with different disease progression (**C**). The binding sequence diagram of circRNA_SLC8A1 and miR-20b-5p (**D**). HEK-293 T cells were co-transfected with circRNA_SLC8A1-wild type or circRNA_SLC8A1-mutant and NC mimic or miR-20b-5p mimic. Firefly and Renilla luciferase activities were determined (**E**). The macrophages were transfected with circRNA_SLC8A1 or sh-circRNA_SLC8A1 or its corresponding control, and then infected with 5 MOI of Mtb for 12 h. The expression of circRNA_SLC8A1 was detected with RT-qPCR (**F**). The macrophages were transfected with circRNA_SLC8A1 or/and miR-20b-5p mimic, and then infected with 5 MOI of Mtb for 12 h. The expression of miR-20b-5p was detected with RT-qPCR (**G**). The expression of miR-20b-5p in Mtb-infected macrophages with different MOI was detected with RT-qPCR (**H**). The macrophages were infected with Mtb for 0, 12, 24 and 48 h, respectively, and the expression of miR-20b-5p in macrophages was detected with RT-qPCR. Mtb: Mycobacterium tuberculosis. Data were shown as mean ± SEM of one representative experiment, similar results were obtained from three independent experiments. N = 5, *P < 0.05, **P < 0.01.

**Figure 2:**
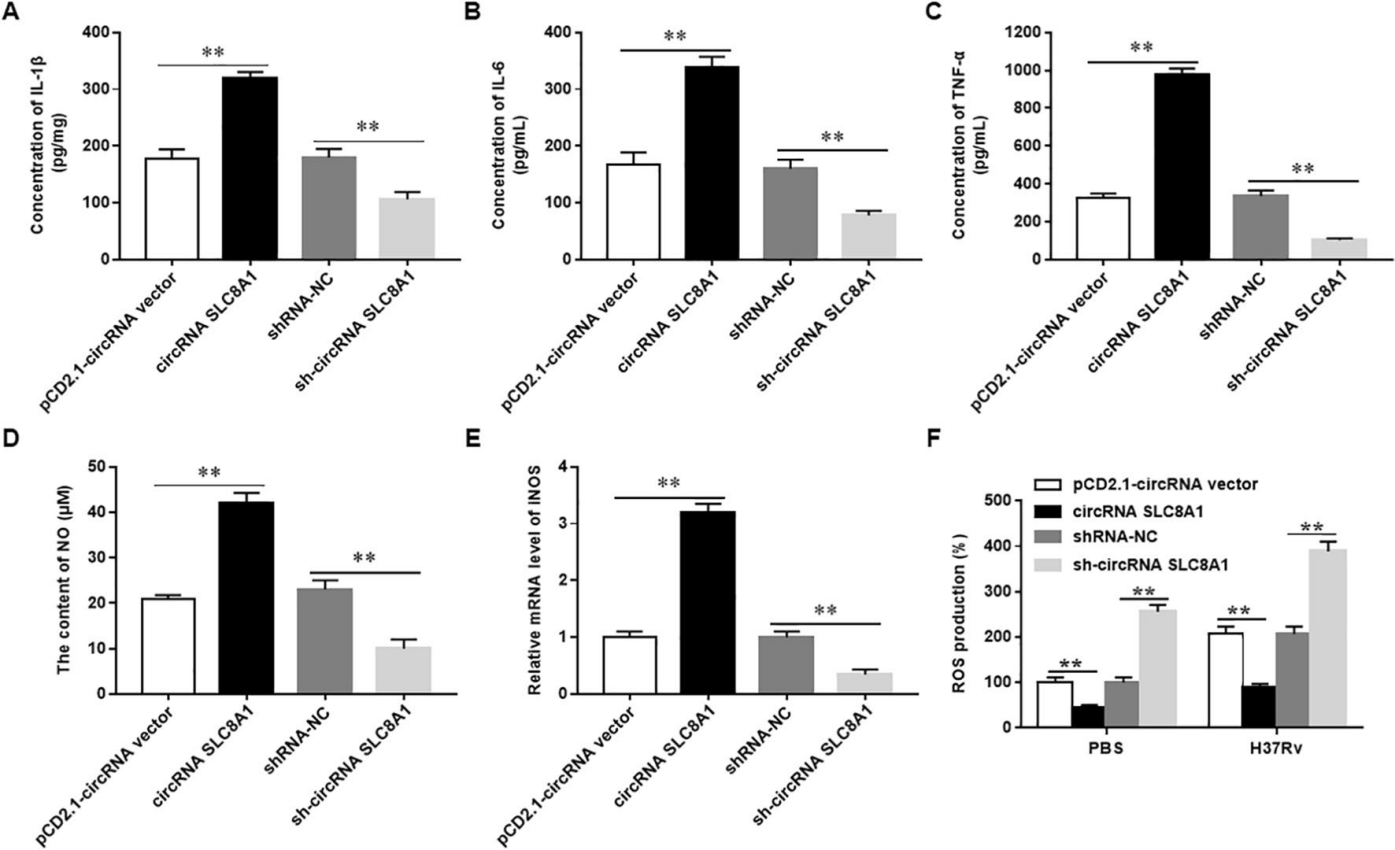
Overexpression of circRNA_SLC8A1 promotes the expression of inflammatory mediators and inhibits ROS production in Mtb-infected macrophages. The macrophages were transfected with circRNA_SLC8A1 or sh-circRNA_SLC8A1 or its corresponding control, and then infected with 5 MOI of Mtb or PBS for 12 h (**A-C**). The concentrations of IL-1β (**A**), IL-6 (**B**) and TNF-α (**C**) in the supernatant were measured with ELISA. (**D**) The content of NO was detected with Griess assay (**E**). The mRNA level of iNOS was measured with RT-qPCR (**F**). The production of ROS was measured with the flow cytometry. Mtb: Mycobacterium tuberculosis. Data were shown as mean ± SEM of one representative experiment, similar results were obtained from three independent experiments. N = 5, *P < 0.05, **P < 0.01.

Total RNA was extracted by using TRIzol reagent (Invitrogen, Shanghai). For circ_SLC8A1, SQSTM1/p62, and iNOS expression, cDNA was synthesized by using a random hexamer with a PrimeScript RT Reagent Kit (Takara, Dalian, China), and Real-time quantitative PCR analyses were conducted with a Platinum™ Taq DNA Polymerase High Fidelity kat (Thermo Fisher Scientific, Shanghai, China). For miR-20b-5p expression, cDNA was synthesized by using miR-20b-5p stem-loop primers or U6-specific primers with a TaqMan™ MicroRNA reverse transcription kit (Thermo Fisher Scientific, Shanghai, China). Real-time quantitative PCR analyses were conducted with a TaqMan Universal Master Mix II kit (Thermo Fisher Scientific, Shanghai, China). RT-qPCR was carried out on a Biosystems 7500 Real-Time PCR System (Applied Biosystems, Foster City, CA), following the thermal cycling procedures: 95 °C for 10 min and 34 cycles of 95 °C for 15 s and 55 °C for 30. Then the melting and dissociation cycling procedures were carried out: 95 °C for 5 s and 60 °C for 5 s, and this procedure continued until the temperature reached 95 °C, increasing the temperature of one cycle by 0.5 °C. U6 and β-actin was used as an endogenous control, and the details of primers were listed in Table 3. The relative expressions of target genes were calculated based on Ct values compared to a reference gene by using the 2^−ΔΔCT^ method. In Figs. 1A,B,E–H, 2E, 4A,D, 6C, 7A,G, 8A,B, the amplification efficiency and R^2^ were: E = 98.7%, R^2^ = 0.981; E = 99.1%, R^2^ = 0.983; E = 98.9%, R^2^ = 0.982; E = 99.3%, R^2^ = 0.985; E = 99.2%, R^2^ = 0.984; E = 98.3%, R^2^ = 0.979; E = 99.5%, R^2^ = 0.987; E = 99.3%, R^2^ = 0.986; E = 98.9%, R^2^ = 0.982; E = 99.6%, R^2^ = 0.988; E = 98.7%, R^2^ = 0.978; E = 99.4%, R^2^ = 0.983; E = 99.6%, R^2^ = 0.988; E = 98.8%, R^2^ = 0.984.

**Table 3:** The primers used for RT-qPCR.

Genes	Forward primer (5'–3')	Reverse primer (5'–3')
pri-miR-20b-5p	AGAGGATAAGATTGGGTCCTA	ACAAGAGATTTGTTATCCAAGA
miR-20b-5p	GCGCAAAGTGCTCATAGTGC	AGTGCAGGGTCCGAGGTATT
miR-20b-5p RT	5′-GTCGTATCCAGTGCAGGGTCCGAGGTATTCGCACTGGATACGACCTACCT-3′	
U6	CTCGCTTCGGCAGCACA	AACGCTTCACGAATTTGCGT
circ_SLC8A1	ATCGAAGGGACTGCCAGAGG	GGTGAAAGACTTAATCGCCGC
SQSTM1/p62	AGGCGCACTACCGCGAT	CGTCACTGGAAAAGGCAACC
iNOS	TGACCATCATGGACCACCAC	ACCAGCCAAATCCAGTCTGC
β-actin	TACCTCATGAAGATCCTCACC	TTTCGTGGATGCCACAGGAC

### Statistical analysis

All data were presented as mean ± SEM. Statistical analysis was performed with SPSS version 22.0 software. Data distribution normality and homogeneity of variance were assessed using the Shapiro–Wilk test and Levene test, respectively. One way analysis of variance (ANOVA) followed by Tukey HSD test were applied for evaluating the significance among multiple groups, and Student’s *t*-test was performed to assess the significance between two groups, according to the data normal distribution and homogeneity of variances. The significance level was set as *P* < 0.05.

### Ethics approval

This study was approved by the Ethics Committee of the First Affiliated Hospital of Xinxiang Medical University.

## Results

### circRNA_SLC8A1 as a sponge of miR-20b-5p is upregulated in tuberculosis patients

One previous microarray analysis found that 32 circRNAs were upregulated in human monocyte derived macrophages response to Mtb infection, and circRNA_SLC8A1 was found to be one of the upregulated circRNAs^18^. To validate microarray analysis findings, we detected the expression of circRNA_SLC8A1 in the peripheral blood of 126 tuberculosis patients (TB), including 51 cases of active pulmonary tuberculosis (PTB), 33 cases of latent pulmonary tuberculosis infection (LPTB), and 42 cases of extra-pulmonary tuberculosis infection (EPTB), and 49 healthy volunteers (HC). The results showed that circRNA_SLC8A1 expression was upregulated in the peripheral blood of PTB, LPTB, and EPTB patients, especially PTB (Fig. 1A). Moreover, we found that circRNA_SLC8A1 was significantly upregulated with the severity of the disease in PTB patients (Fig. 1B). Next, we predicated the potential targeting miRNAs of circRNA_SLC8A1 by using StarBase and verified that circRNA_SLC8A1 bound to miR-20b-5p (Fig. 1C,D)). Overexpression of circRNA_SLC8A1 inhibited miR-20b-5p expression, and overexpressing miR-20b-5p counteracted the inhibitory effect of circRNA_SLC8A1 overexpressing on miR-20b-5p expression (Fig. 1E,F)). Furthermore, we found that miR-20b-5p expression was decreased in a time and dose-dependent manner in macrophages in response to Mtb infection (Fig. 1G,H) . Meanwhiles, we also detected the expression level of pri-miR-20b-5p in the peripheral blood of HC and PTB patients, as well as in Mtb infected macrophages. The results showed that Mtb infection had no effect on the expression level of pri-miR-20b-5p (Supplementary Fig. 1). These results indicated that sponging of miR-20b-5p by circRNA_SLC8A1 could serve as a mechanism for the diminished levels of miR-20b-5b in Mtb infected patients.

### Overexpression of circRNA_SLC8A1 promotes the expression of inflammatory mediators and inhibits ROS production in Mtb-infected macrophages

When invading mycobacteria are detected, macrophages produce NO and various cytokines to activate the adaptive immune response, thereby combating tuberculosis infection^23^. To explore the regulatory of circRNA_SLC8A1 in Mtb-infected macrophages, we transfected Mtb-infected macrophages with circRNA_SLC8A1 overexpression vector (circRNA_SLC8A1) to observe the production of inflammation mediators and ROS in Mtb-infected macrophages. As shown in Fig. 2, overexpression of circRNA_SLC8A1 significantly promoted the secretion of IL-1β, IL-6, TNF-a, increased NO content, and induced the mRNA expression of iNOS in Mtb-infected U937 cells, whereas silencing circRNA_SLC8A1 significantly reduced the secretion of IL-1β, IL-6, and TNF-a and decreased NO content and expression of iNOS mRNA (Fig. 2A–E). ROS are important microbicidal mediators of macrophages and directly kill mycobacteria^24^. Therefore, we tested the effects of circRNA_SLC8A1 overexpressing or silencing on the production of ROS. Macrophages were transfected with circRNA_SLC8A1 or sh-circRNA_SLC8A1, and infected with or without H37Ra. Flow cytometry showed that the levels of ROS were downregulated by transfection with circRNA_SLC8A1 in U937 cells (Fig. 2F). These results showed that overexpression of circRNA_SLC8A1 promoted the expression of inflammatory mediators and inhibited ROS production in Mtb-infected macrophages.

### Knock-down of circRNA_SLC8A1 induces the apoptosis of Mtb-infected macrophages and inhibits the survival of Mtb in macrophages

**Figure 3:**
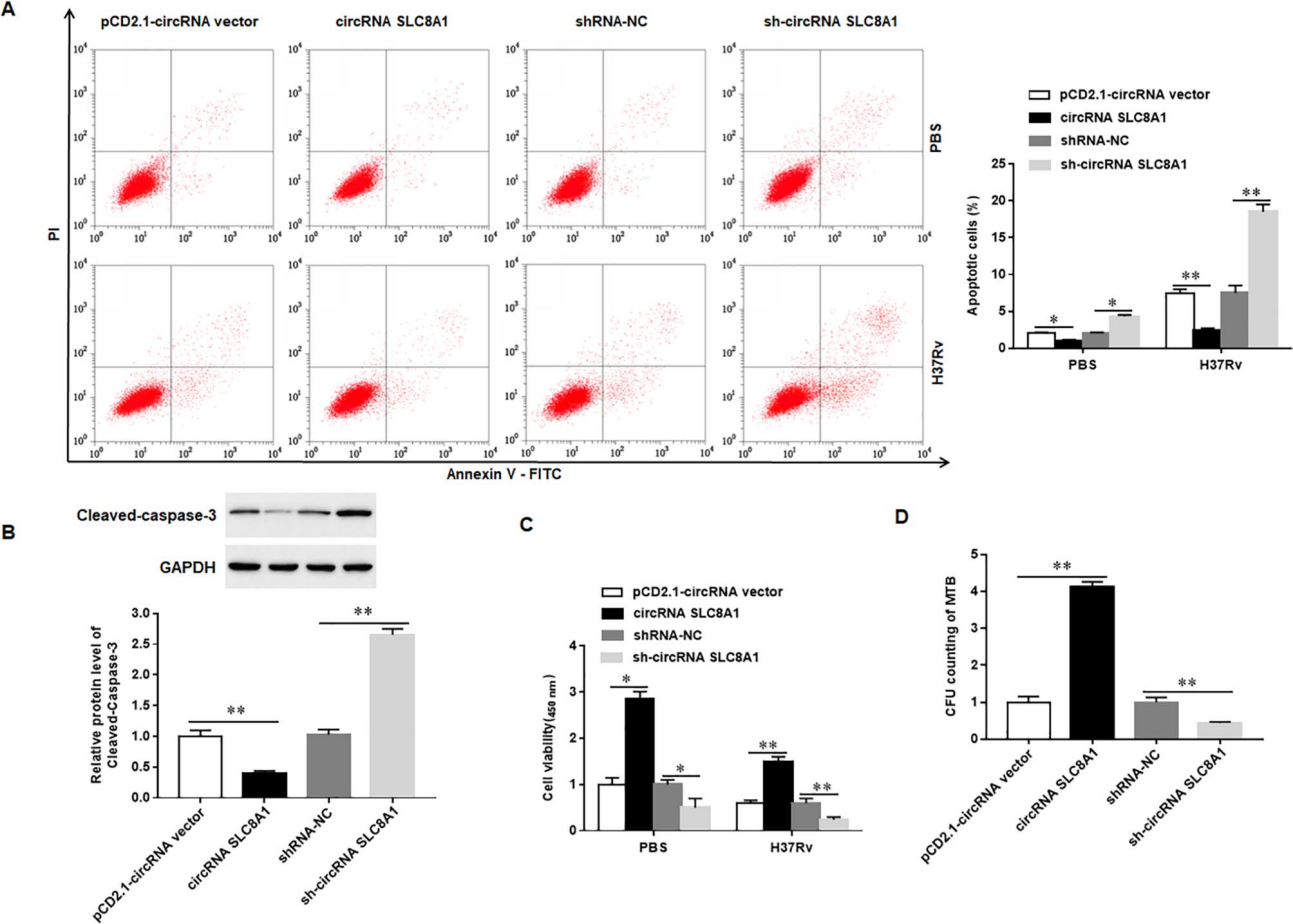
Knock-down of circRNA_SLC8A1 induces the apoptosis of Mtb-infected macrophages and inhibits the survival of Mtb in macrophages. The macrophages were transfected with circRNA_SLC8A1 or sh-circRNA_SLC8A1 or its corresponding control, and then infected with 5 MOI of Mtb or PBS for 12 h (**A**). The apoptosis of macrophage**s** was measured with the flow cytometry (**B**). The protein levels of Cleaved-caspase-3 were detected with Western blotting (**C**). The viability of macrophage**s** was measured with CCK-8 (**D**). The survival of Mtb in macrophages was measured with CFU assay. Mtb: Mycobacterium tuberculosis. Data were shown as mean ± SEM of one representative experiment, similar results were obtained from three independent experiments. N = 5, *P < 0.05, **P < 0.01.

When Mtb infects macrophages, macrophages typically undergo both necrotic and apoptotic forms of death; necroptosis is one mechanism by which bacteria can egress from macrophages, evade host defense, and spread. Conversely, apoptosis of macrophages is associated with reduced pathogen survival^25^. We transfected circRNA_SLC8A1 or sh-circRNA_SLC8A1 into Mtb-infected macrophages and observed the apoptosis of macrophages and the survival of Mtb in macrophages. The results showed that overexpression of circRNA_SLC8A1 significantly reduced apoptosis of macrophages and deceased the protein expression of Cleaved-caspase-3, whereas silencing circRNA_SLC8A1 significantly induced apoptosis of macrophages and increased Cleaved-caspase-3 protein expression (Fig. 3A,B). Treatment with Ac-DEVD-CHO, a specific caspase-3 inhibitor, markedly reversed the induction of circRNA_SLC8A1 silencing on cell apoptosis (Supplementary Fig. 2). Furthermore, we also observed that overexpression of circRNA_SLC8A1 increased the viability of macrophages and promoted the survival of Mtb, and silencing circRNA_SLC8A1 significantly deceased the viability of macrophages and inhibited the survival of Mtb in macrophages (Fig. 3C,D). These results indicated that silencing circRNA_SLC8A1 induced the apoptosis of Mtb-infected macrophages and inhibited the survival of Mtb in macrophages.

### Overexpression of miR-20b-5p inhibits the expression of inflammatory mediators and promotes ROS production in Mtb-infected macrophages

**Figure 4:**
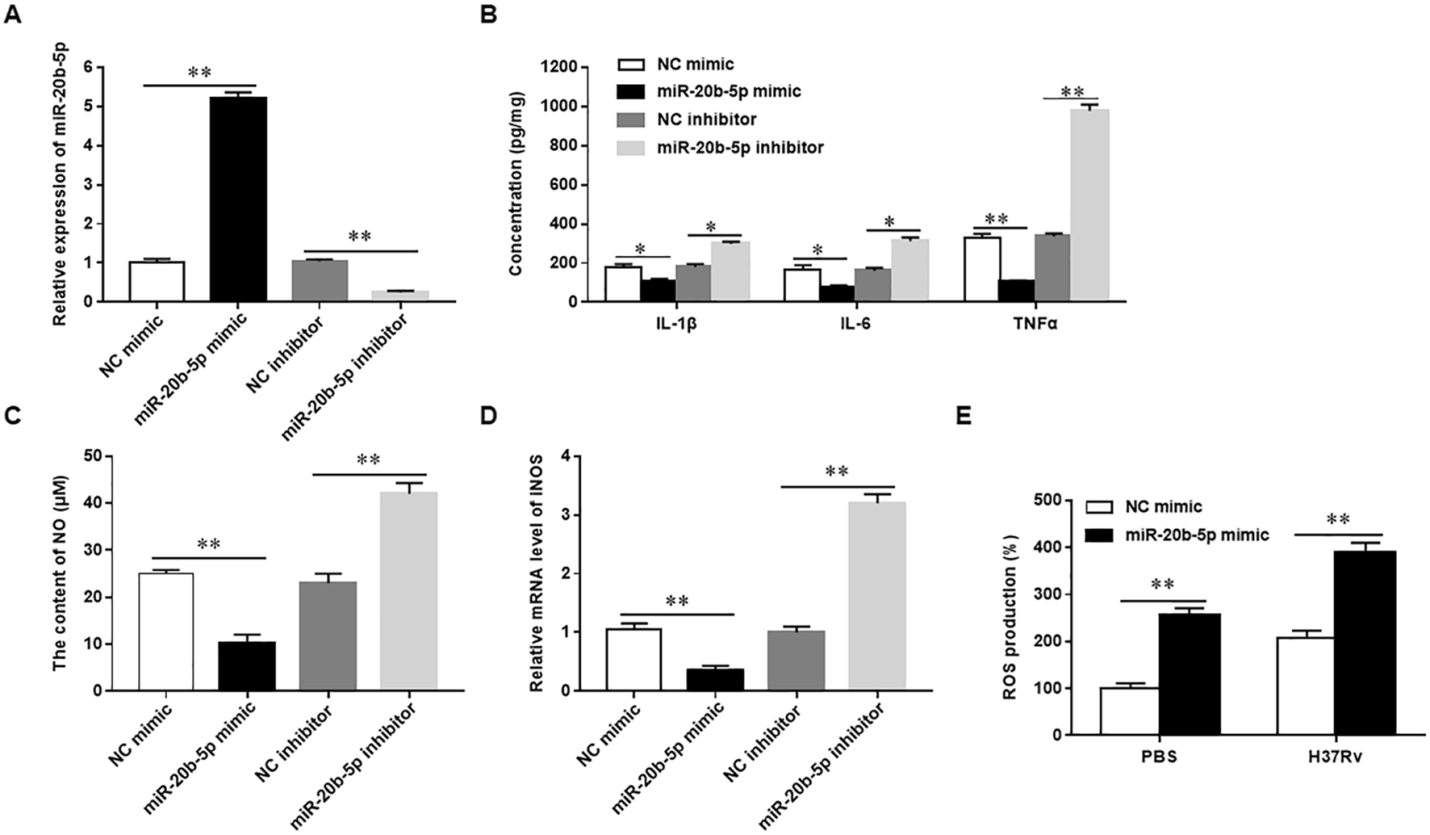
Overexpression of miR-20b-5p inhibits the expression of inflammatory mediators and promotes ROS production in Mtb-infected macrophages. The macrophages were transfected with miR-20b-5p mimic or inhibitor, and then infected with 5 MOI of Mtb or PBS for 12 h (**A**). The expression of miR-20b-5p was detected with RT-qPCR (**B**). The concentrations of IL-1β, IL-6 and TNF-α in the supernatant were measured with ELISA. **C**. The content of NO was detected with Griess assay. **D**. The mRNA level of iNOS was measured with RT-qPCR.** E**. The production of ROS in macrophages was measured with the flow cytometry. Mtb: Mycobacterium tuberculosis. Data were shown as mean ± SEM of one representative experiment, similar results were obtained from three independent experiments. N = 5, *P < 0.05, **P < 0.01.

To explore whether miR-20b-5p plays a role in Mtb infected macrophages, U937 cells were transfected with miR-20b-5p mimic and miR-20b-5p inhibitor and their negative controls for 24 h, and then infected with Mtb at 5 MOI for 12 h. We observed that overexpression of miR-20b-5p significantly inhibited the secretion of IL-1β, IL-6 and TNF-a, decreased the contents of NO and mRNA expression of iNOS, and promoted the production of ROS in Mtb-infected U937 cells. However, transfection of Mtb-infected U937 cells with miR-20b-5p inhibitor significantly induced the secretion of IL-1β, IL-6 and TNF-a and increased the contents of NO and mRNA expression of iNOS (Fig. 4A–E). These results demonstrated that miR-20b-5p inhibited the expression of inflammatory mediators and promoted ROS production in Mtb-infected macrophages.

### Overexpression of miR-20b-5p reverses the effects of circRNA_SLC8A1 on Mtb-infected macrophages

**Figure 5:**
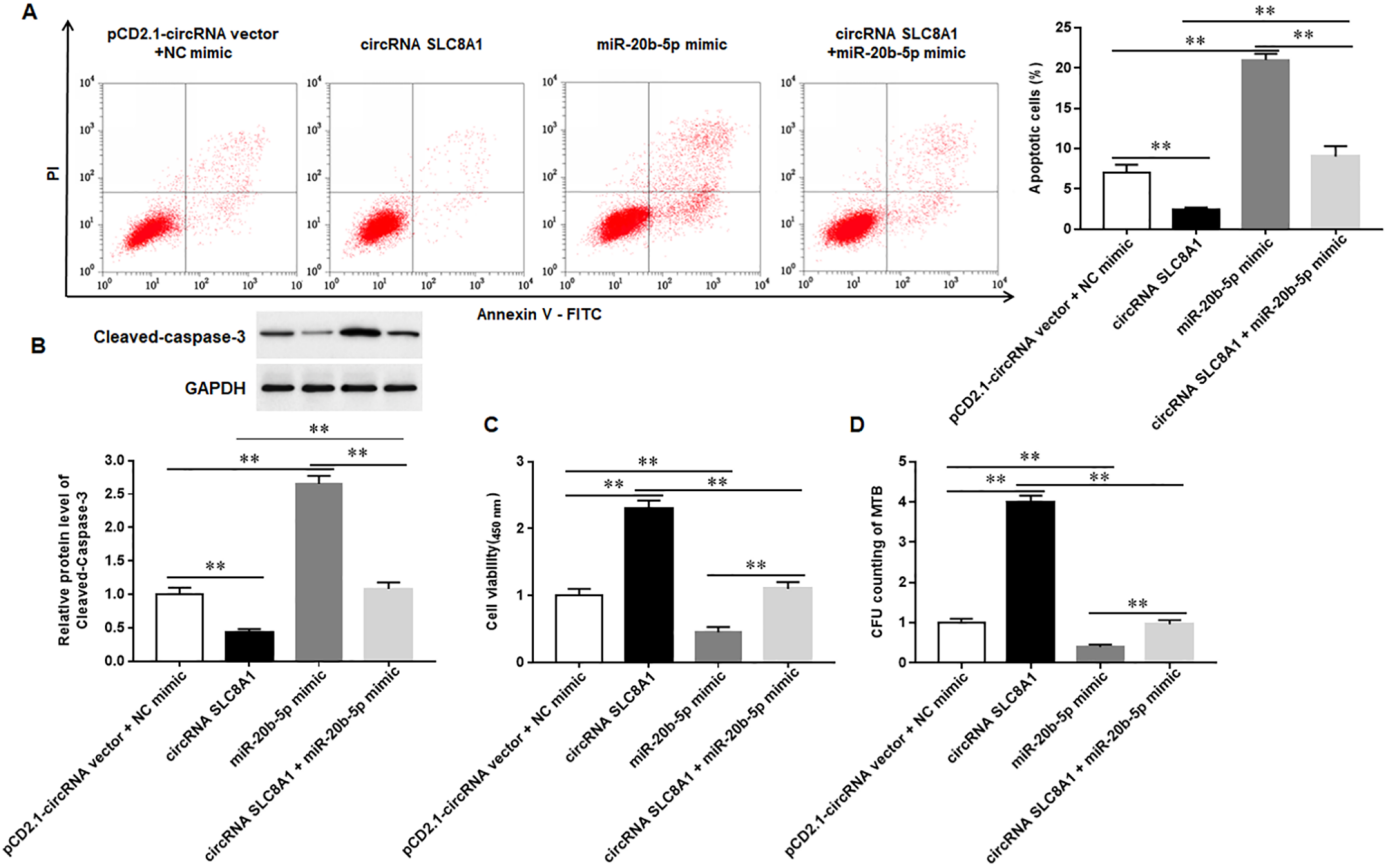
circRNA_SLC8A1 inhibits the apoptosis of Mtb-infected macrophages by targeting miR-20b-5p. The macrophages were transfected with circRNA_SLC8A1 overexpression vector (circRNA_SLC8A1) and/or miR-20b-5p mimic, and then infected with 5 MOI of Mtb for 12 h (**A**). The apoptosis of macrophage**s** was measured with flow cytometry (**B**). The protein levels of Cleaved-caspase-3 were detected with Western blotting (**C**). The viability of macrophage**s** was measured with CCK-8 (**D**). The survival of Mtb in macrophages was measured with CFU assay. Mtb: Mycobacterium tuberculosis. Data were shown as mean ± SEM of one representative experiment, similar results were obtained from three independent experiments. N = 5, *P < 0.05, **P < 0.01.

We then investigated the effects of miR-20b-5p on Mtb-infected macrophages transfected with circRNA_SLC8A1 overexpression vector. The results showed that overexpression of circRNA_SLC8A1 inhibited cell apoptosis and decreased the protein expression of Cleaved-caspase-3 in Mtb-infected macrophages, whereas overexpression of miR-20b-5p significantly reversed the inhibitory effects of circRNA_SLC8A1 overexpression on the apoptosis and Cleaved-caspase-3 protein expression in Mtb-infected macrophages. In addition, miR-20b-5p mimics were transfected into Mtb-infected macrophages, and which significantly decreased the viability of macrophages and inhibited the survival of Mtb in macrophages. Co-transfection of circRNA_SLC8A1 and miR-20b-5p mimic into Mtb-infected macrophages significantly increased the viability of macrophages and promoted the survival of Mtb in macrophages compared with macrophages transfected with miR-20b-5p mimics (Fig. 5C,D). The above results suggested that circRNA_SLC8A1 inhibited the apoptosis of Mtb-infected macrophages and promoted the survival of Mtb in macrophages through targeting miR-20b-5p.

### miR-20b-5p targets the 3’-UTR of SQSTM1/p62 and inhibits its expression post transcriptionally

**Figure 6:**
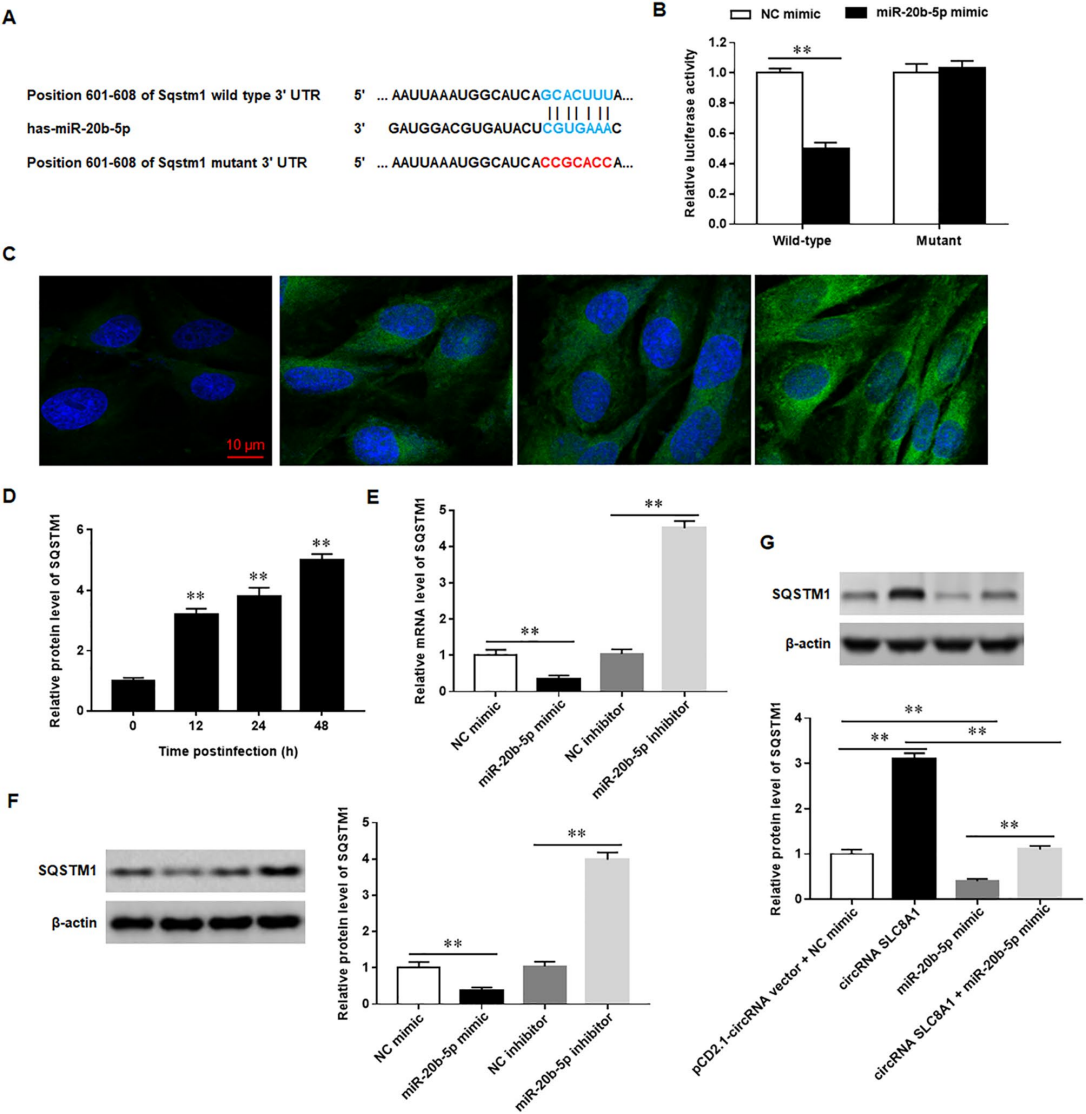
miR-20b-5p targets the 3’-UTR of SQSTM1/p62 and inhibits its expression post transcriptionally.(**A**) The binding sequence diagram of miR-20b-5p and SQSTM1/p62 (**B**) HEK-293 T cells were co-transfected with SQSTM1/p62-wild type or SQSTM1/p62-mutant and NC mimic or miR-20b-5p mimic. Firefly and Renilla luciferase activities were determined (**C-D**). The macrophages were infected with Mtb at 5 MOI for 0, 12, 24 and 48 h, respectively, and the protein levels of SQSTM1/p62 were detected with immunofluorescence analysis (**E**). The macrophages were transfected with miR-20b-5p mimic or miR-20b-5p inhibitor or its corresponding control, and then infected with 5 MOI of Mtb for 12 h. The mRNA (**E**) and protein (**F**) levels of SQSTM1/p62 were detected with RT-qPCR and Western blotting, respectively (**G**). The macrophages were transfected with circRNA_SLC8A1 overexpression vector (circRNA_SLC8A1) and/or miR-20b-5p mimic, and then infected with 5 MOI of Mtb for 12 h. The protein levels of SQSTM1/p62 were detected with Western blotting. Mtb: Mycobacterium tuberculosis. Data were shown as mean ± SEM of one representative experiment, similar results were obtained from three independent experiments. N = 5, compared with 0 h *P < 0.05, **P < 0.01.

To determine the regulatory mechanism of miR-20b-5p in cell apoptosis and the expression of inflammatory cytokines, bioinformatics analysis was performed by using StarBase (http://starbase.sysu.edu.cn) to predict the downstream targets of miR-20b-5p. SQSTM1/p62 displayed a potential seed match for miR-20b-5p in its untranslated region (UTR) (Fig. 6A). Next, we performed the luciferase reporter gene assay to validate the binding of miR-20b-5p to SQSTM1/p62. The results indicated that miR-20b-5p directly bound to SQSTM1/p62 3’-UTR. Furthermore, the expression of SQSTM1/p62 in macrophages was dose dependently upregulated with Mtb infection time (Fig. 6C,D). Overexpression of miR-20b-5p significantly downregulated the mRNA and protein expression of SQSTM1/p62, while miR-20b-5p inhibitor promoted the mRNA and protein expression of SQSTM1/p62 (Fig. 6 E and F). Meanwhile, we also found that overexpression of circRNA_SLC8A1 substantially increased the protein levels of SQSTM1/p62, while overexpression of miR-20b-5p offset the promoting of circRNA_SLC8A1 overexpressing on SQSTM1/p62 protein expression (Fig. 6G). These results demonstrated that SQSTM1/p62 was a target of miR-20b-5p, and SQSTM1/p62 expression was negatively modulated by miR-20b-5p.

### Overexpression of SQSTM1/p62 promotes the expression of inflammatory mediators and activates the NF-κB pathway in Mtb-infected macrophages

**Figure 7:**
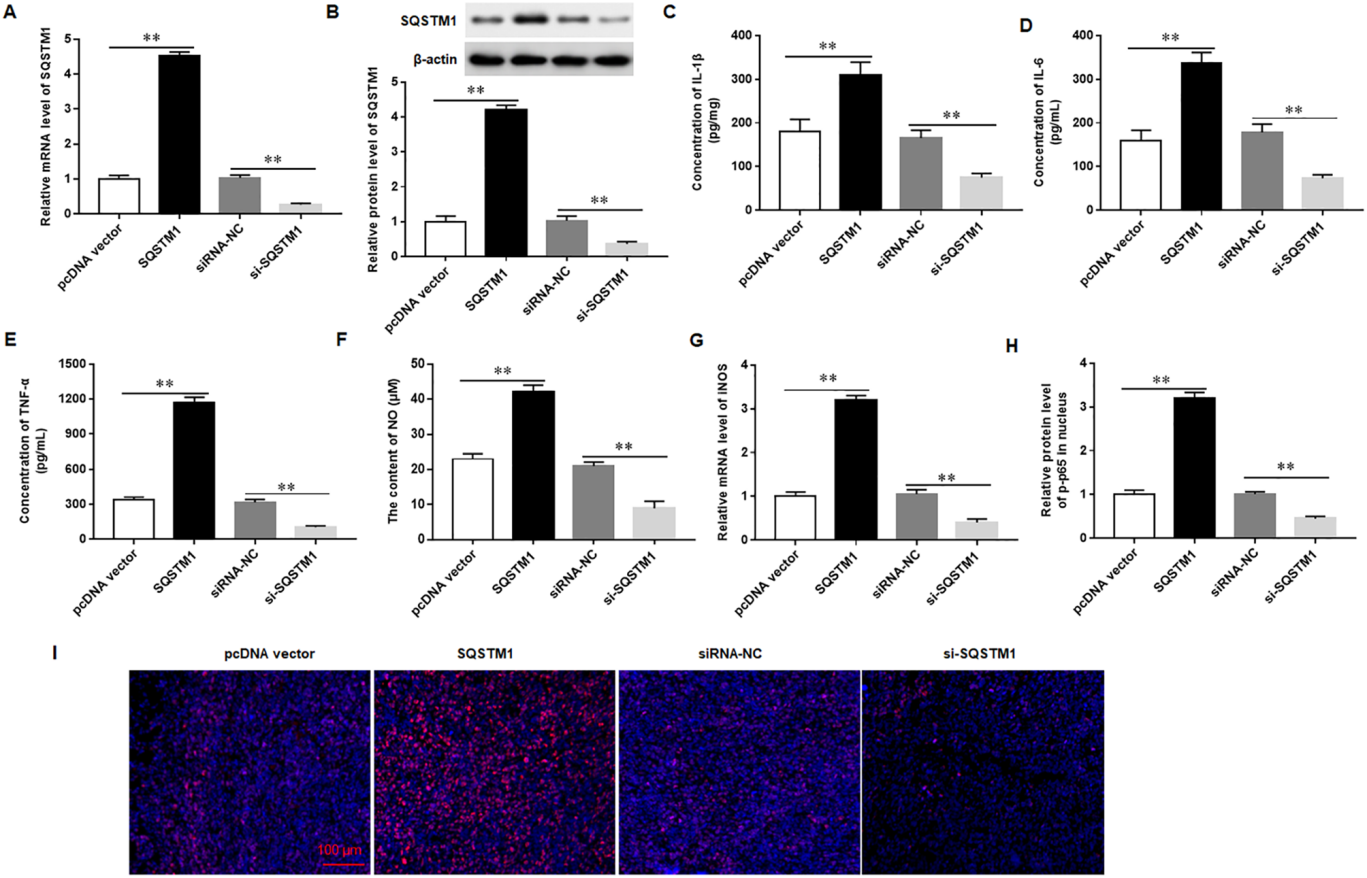
Overexpression of SQSTM1/p62 promotes the expression of inflammatory mediators and activates the NF-κB pathway in Mtb-infected macrophages.The macrophages were transfected with SQSTM1/p62 overexpression vector (SQSTM1/p62) or SQSTM1/p62 siRNA (si-SQSTM1/p62), and then infected with 5 MOI of Mtb for 12 h (**A-B**). The mRNA (**A**) and protein (**B**) expression of SQSTM1/p62 was detected with RT-qPCR and Western blotting, respectively (**C-E**) The concentrations of IL-1β (**C**), IL-6 (**D**) and TNF-α (**E**) in the supernatant were measured with ELISA (**F**). The content of NO was detected with Griess assay (**G**). The mRNA level of iNOS was measured with RT-qPCR (**H-I**). The protein levels of p-NF-κB(p-p65) in nuclear of macrophages were measured with Immunofluorescence analysis. Mtb: Mycobacterium tuberculosis. Data were shown as mean ± SEM of one representative experiment, similar results were obtained from three independent experiments. N = 5, *P < 0.05, **P < 0.01.

To investigate the role of SQSTM1/p62 in Mtb-infected macrophages, SQSTM1/p62 overexpression vector and siRNA were transfected into Mtb-infected macrophages. The results showed that overexpression of SQSTM1/p62 markedly promoted the secretion of IL-1β, IL-6 and TNF-a, increased the contents of NO, and promoted the mRNA expression of iNOS in Mtb-infected U937 cells. Silencing SQSTM1/p62 reduced the secretion of IL-1β, IL-6 and TNF-a, decreased the contents of NO, and inhibited iNOS mRNA expression (Fig. 7A–G). In addition, we also found that overexpression of SQSTM1/p62 increased p-p65 protein levels in the nucleus whereas silencing SQSTM1/p62 inhibited p-p65 protein expression in the nucleus (Fig. 7H,I). These results demonstrated that SQSTM1/p62 promoted the expression of inflammatory mediators and activation of the NF-κB pathway in Mtb-infected macrophages.

### miR-20b-5p targets SQSTM1/p62 to promote macrophages to resist Mtb infection through the NF-kB signaling pathway

**Figure 8:**
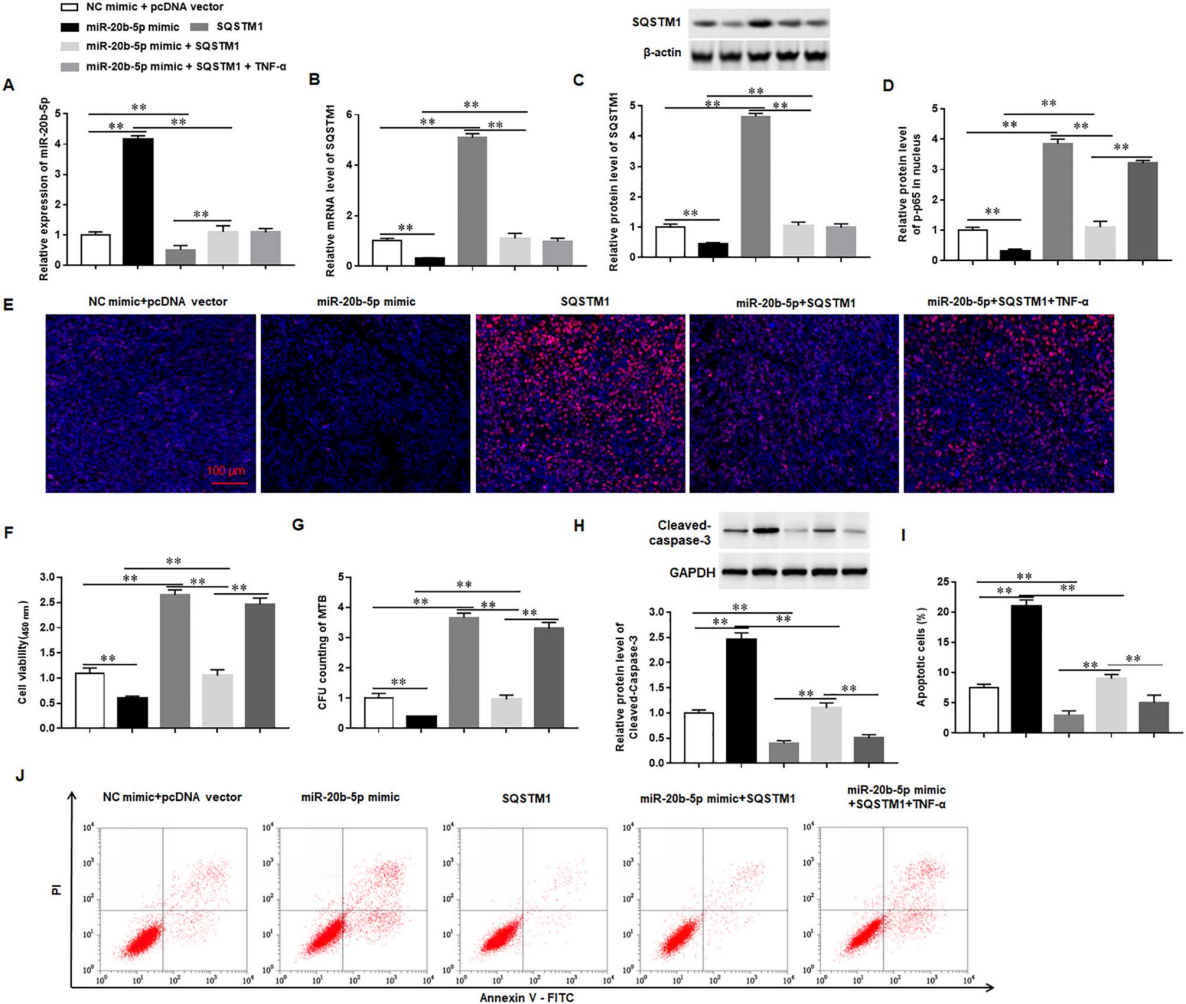
miR-20b-5p targets SQSTM1/p62 to promote macrophages to resist Mtb infection through the NF-kB signaling pathway. The macrophages were transfected with miR-20b-5p mimic and/or SQSTM1/p62 overexpression vector (SQSTM1/p62) and treated with or without TNF-α, and then infected with Mtb for 12 h (**A**). The expression of miR-20b-5p was detected with RT-qPCR (**B**-**C**). The mRNA (**B**) and protein (**C**) expression levels of SQSTM1/p62 were detected with RT-qPCR and Western blotting, respectively (**D**-**E**). The protein levels of p-NF-κB (p-p65) in nuclear of macrophages were measured with Immunofluorescence analysis. (**F**) The viability of macrophages was measured with CCK-8 (**G**). The survival of Mtb in macrophages was measured with CFU assay (**H**). The protein levels of Cleaved-caspase-3 were detected with Western blotting. (**I**-**J**) The apoptosis of macrophages was measured with the flow cytometry. Mtb: Mycobacterium tuberculosis. Data were shown as mean ± SEM of one representative experiment, similar results were obtained from three independent experiments. N = 5, *P < 0.05, **P < 0.01.

Previous reports have shown that the NF-κB pathway was activated in macrophages infected with Mtb to induce the expression of inflammatory cytokines^26,27^. Although our results suggested that SQSTM1/p62 promotes expression of p-p65 protein in nucleus of Mtb-infected macrophages, it is still unclear whether miR-20b-5p, as an upstream target of SQSTM1/p62, regulates the NF-κB pathway. We transfected Mtb-infected macrophages with miR-20b-5p mimic and/or SQSTM1/p62 overexpression vector and/or TNF-α, an activator of the NF-κB pathway. The results showed that overexpression of miR-20b-5p inhibited p-p65 protein expression in the nucleus, while overexpression of SQSTM1/p62 reversed the inhibitory effect of miR-20b-5p on the expression of p-p65 protein. Whereas TNF-α had no effects on the expression of miR-20b-5p and SQSTM1/p62, promoting activation of the NF-κB pathway (Fig. 8A–E). Moreover, we found that overexpression of miR-20b-5p decreased the viability of macrophages, inhibited the survival of Mtb in macrophages, and promoted cell apoptosis and Cleaved-caspase-3 protein expression, while overexpression of SQSTM1/p62 increased the viability of macrophages and the survival of Mtb in macrophages and inhibited cell apoptosis and Cleaved-caspase-3 protein expression. TNF-α increased the viability of macrophages, promoted the survival of Mtb in macrophages, reduced cell apoptosis and decreased Cleaved-caspase-3 protein expression (Fig. 8F–J). These results suggested that miR-20b-5p targeted SQSTM1/p62 to inhibit the survival of Mtb and promote the apoptosis of macrophages through blocking the NF-kB pathway.

**Figure 9:**
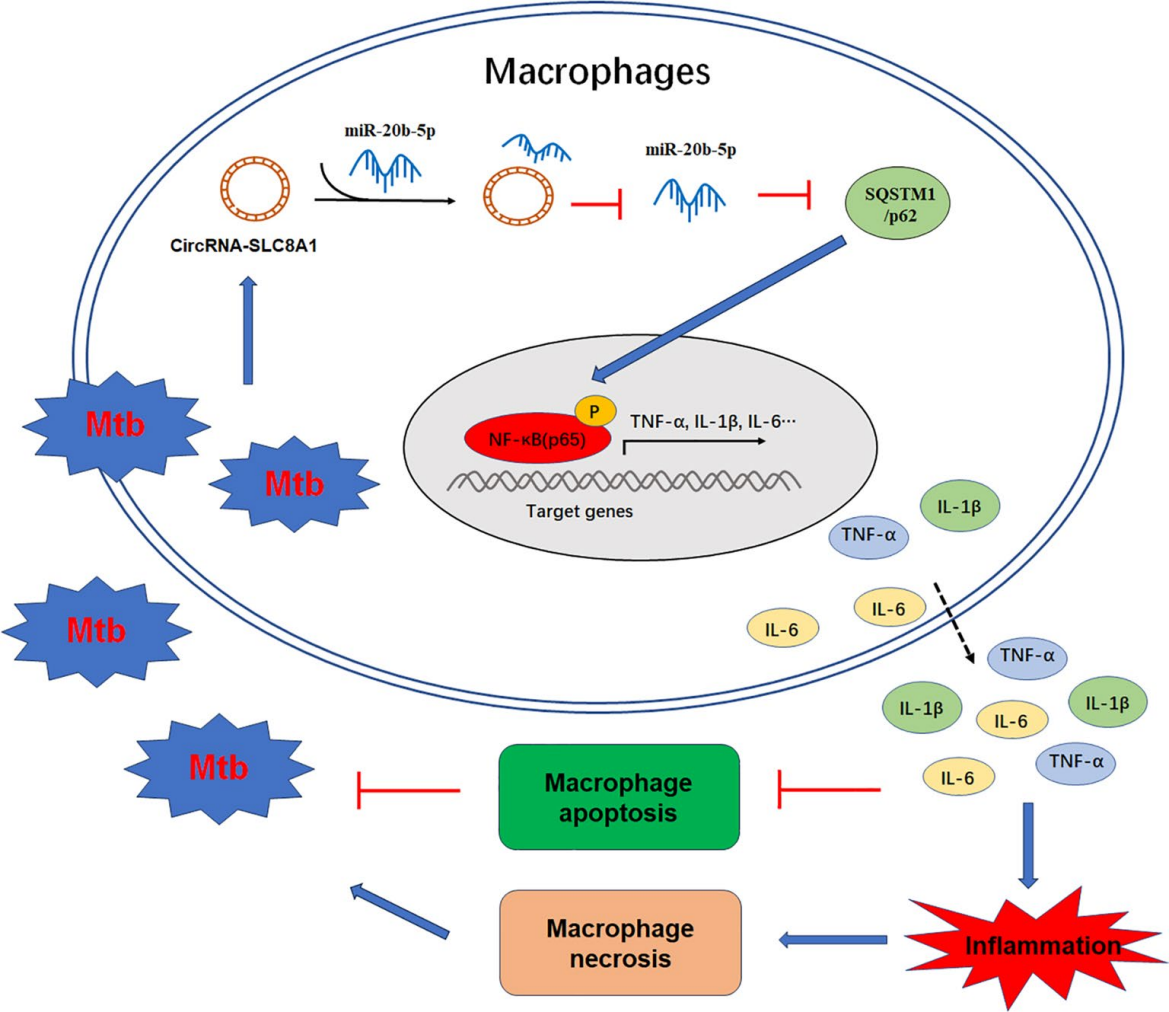
The mechanistic schematic model of action of circRNA_SLC8A1 in macrophages against Mtb infection. Briefly, circRNA_SLC8A1 sponged miR‐20b‐5p to upregulate SQSTM1/p62 expression, further activating the NF-κB signaling pathway to inhibit macrophage apoptosis and promote Mtb survival in macrophages.

In summary, Mtb infection leads to the upregulation of circRNA_SLC8A1 in macrophages, and circRNA_SLC8A1 upregulates SQSTM1/p62 expression by sponging miR‐20b‐5p. Upregulated SQSTM1/p62 promotes the secretion of inflammatory mediators by activating the NF-κB signaling pathway, which in turn reduces macrophage apoptosis and promotes the survival of Mtb in macrophages, ultimately leading to the occurrence and progression of pulmonary tuberculosis (Fig. 9).

## Discussion

Tuberculosis is one of the most common chronic infectious diseases in the world, causing a serious threat to public health. Tuberculosis has a complex pathogenesis, and the clinical symptoms are atypical, making it difficult to diagnose early. Macrophage apoptosis plays a crucial role in Mtb clearance in pulmonary tuberculosis disease. In the present study, we probed the role of circRNA_SLC8A1 in Mtb-infected macrophage apoptosis and Mtb survival in macrophages and results demonstrated that circRNA_SLC8A1 was upregulated in Mtb-infected macrophages, which is consistent with a previous report^18^. Furthermore, we also found that overexpressing circRNA_SLC8A1 promoted the secretion of inflammatory mediators IL-1β, IL-6, TNF-α, and NO, increased INOS expression, reduced ROS production, inhibited the apoptosis of Mtb-infected macrophages and Cleaved-caspase-3 protein expression, and promoted the survival of Mtb in macrophages. From this we identified circRNA_SLC8A1 is involved in macrophage defense against Mtb infection.

circRNAs function by repressing certain miRNAs, which exert further effects by regulating the expression of their downstream target genes^28^. For example, Zhang et al.^29^ reported that circRNA_0078767 was markedly downregulated in both osteosarcoma tissues and cells, and promoted osteosarcoma progression by sponging miRNA-330-3p to upregulate cyclin-dependent kinase 14 (CDK14) expression. Wu et al.’s report^30^ showed that hsa_circ_0045474 sponged miR-582-5p and negatively regulated miR-582-5p expression in Mtb-infected macrophages. miR-582-5p targeted Tankyrase-2 (TNKS2) and inhibited TNKS2 expression. In our study, to explore the regulatory mechanism of circRNA_SLC8A1 in the process of macrophages against Mtb infection, we predicted the downstream targets of circRNA_SLC8A1 by bioinformatics database analysis online. The results showed that circRNA_SLC8A1 as a sponge of miR-20b-5p was upregulated in tuberculosis patients and inhibited miR-20b-5p expression. Whereas miR-20b-5p targeted the 3’-UTR of SQSTM1/p62 and post transcriptionally inhibited SQSTM1/p62 expression.

To explore the role of miR-20b-5p in Mtb-infected macrophages, we transfected Mtb-infected macrophages with overexpression vectors of circRNA_SLC8A1 and/or miR-20b-5p mimic. The results showed that overexpression of miR-20b-5p significantly inhibited the secretion of IL-1β, IL-6 and TNF-a, decreased the contents of NO and mRNA expression of iNOS, and promoted the production of ROS in Mtb-infected U937 cells. Moreover, overexpression of circRNA_SLC8A1 inhibited the apoptosis, decreased Cleaved-caspase-3 protein expression in Mtb-infected macrophages, and promoted the survival of Mtb in macrophages, whereas overexpression of miR-20b-5p significantly reversed the effects of circRNA_SLC8A1 overexpression on Mtb-infected macrophages. Shen et al.^31^ reported that miR-20b-5p was higher expression in M2 macrophages, compared with M1 macrophages. Overexpressing miR-20b-5p suppressed the progression of chronic obstructive pulmonary disease by inhibiting macrophages to M1 phenotype by suppressing inflammatory responses. Zhang et al.^32^ reported that the expression level of miR-20b-5p was downregulated in Mtb-infected macrophages at different times. Overexpression of miR-20b-5p reduced the survival of Mtb in macrophages, decreased the cell viability, and induced cell apoptosis in Mtb-infected macrophages, while silencing miR-20b-5p promoted the survival of Mtb in macrophages, increased the cell vitality and inhibited cell apoptosis in Mtb-infected macrophages. The above reports are consistent with our findings that miR-20b-5p promoted the apoptosis of Mtb-infected macrophages and inhibited the survival of Mtb in macrophages.

SQSTM1/p62 was identified as a potential target for host directed therapy against a broad range of pathogenic bacteria. Lee et al.’s report^33^ showed that chemical mimics of the N-terminal degrons (N-degrons) in proteolytic systems induce autophagic membrane biogenesis and intracellular bacterial recruitment to the autophagic membrane via binding to the ZZ domain of the autophagy receptor SQSTM1/p62, leading to lysosomal degradation. Seto et al.^34^ reported that the autophagy adaptor protein SQSTM1/p62 localized to dendritic cells and mediated autophagosome formation in response to Mtb infection in dendritic cells. In our study, SQSTM1/p62 was dose and time dependently upregulated in macrophages with Mtb infection. Overexpression of SQSTM1/p62 markedly promoted the secretion of IL-1β, IL-6 and TNF-a, increased the contents of NO, and promoted the mRNA expression of iNOS in Mtb-infected macrophages. Furthermore, we found that overexpression of SQSTM1/p62 increased the viability of macrophages, promoted the survival of Mtb in macrophages and inhibited cell apoptosis and Cleaved-caspase-3 protein expression. In addition, we observed that silencing SQSTM1/p62 blocked the NF-kB signaling pathway. This is consistent with previous reports on the NF-kB signaling pathway in Mtb resistance^26,27^.

In summary, we demonstrated that circRNA_SLC8A1 was upregulated in tuberculosis patients, and overexpression of circRNA_SLC8A1 sponged miR‐20b‐5p to upregulate SQSTM1/p62 expression, further activating the NF-κB signaling pathway to promote Mtb survival in macrophages. This study implies circRNA_SLC8A1 as a potential therapeutic target for tuberculosis control.

### Supplementary Information

Supplementary Figures.

## Data Availability

The datasets used during the present study are available from the corresponding author upon reasonable request.
