# Author Correction: circRNA_SLC8A1 promotes the survival of mycobacterium tuberculosis in macrophages by upregulating expression of autophagy-related protein SQSTM1/p62 to activate the NF-κB pathway

**DOI:** 10.1038/s41598-024-59452-2

**Published:** 2024-04-17

**Authors:** Zhenyun Li, Yuan Gao, Bianfang Zhang, Wei Dong, Yuling Xi, Yan Li, Junwei Cui

**Affiliations:** 1 Department of Tuberculosis, The First Affiliated Hospital of Xinxiang Medical University, Weihui, 453100 Henan China; 2 Clinical Pharmacy Office, The First Affiliated Hospital of Xinxiang Medical University, Weihui, 453100 Henan China; 3 Gastrointestinal Surgery, The First Affiliated Hospital of Xinxiang Medical University, Weihui, 453100 Henan China

Correction to: *Scientific Reports* 10.1038/s41598-024-55493-9, published online 04 March 2024

The Funding section in the original version of this article was omitted. The Funding section now reads:

“Funding was provided by Henan Province Science and Technology Research Project [LHGJ20230525].”

The original article has been corrected.

