# Supplementary material for: circRNA_SLC8A1 promotes the survival of mycobacterium tuberculosis in macrophages by upregulating expression of autophagy-related protein SQSTM1/p62 to activate the NF-κB pathway
Source: Sci Rep. 2024 Mar 4;14:5233. doi: 10.1038/s41598-024-55493-9 (PMC10909944; doi:10.1038/s41598-024-55493-9)
Supplement: Supplementary file 1 — Supplementary Figures. [file 41598_2024_55493_MOESM1_ESM.docx]

**Figure legends:**

**
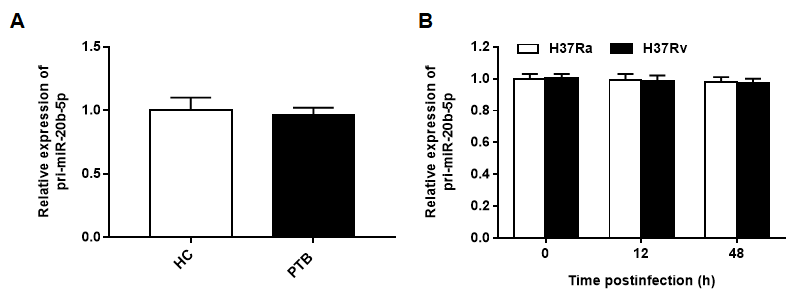
**

**Supplementary Figure 1.** **The expression of pri-miR-20b-5p in PTB patients and Mtb infected macrophages.**

**A**. The expression of pri-miR-20b-5p in the peripheral blood of HC and PTB patients was detected with RT-qPCR. **B.** The expression of pri-miR-20b-5p in Mtb infected macrophages was detected with RT-qPCR. HC: healthy volunteers; PTB: active pulmonary tuberculosis; Mtb: Mycobacterium tuberculosis. Data were shown as mean ± SEM of one representative experiment, similar results were obtained from three independent experiments. N=5, * *P* < 0.05, ** *P* < 0.01.


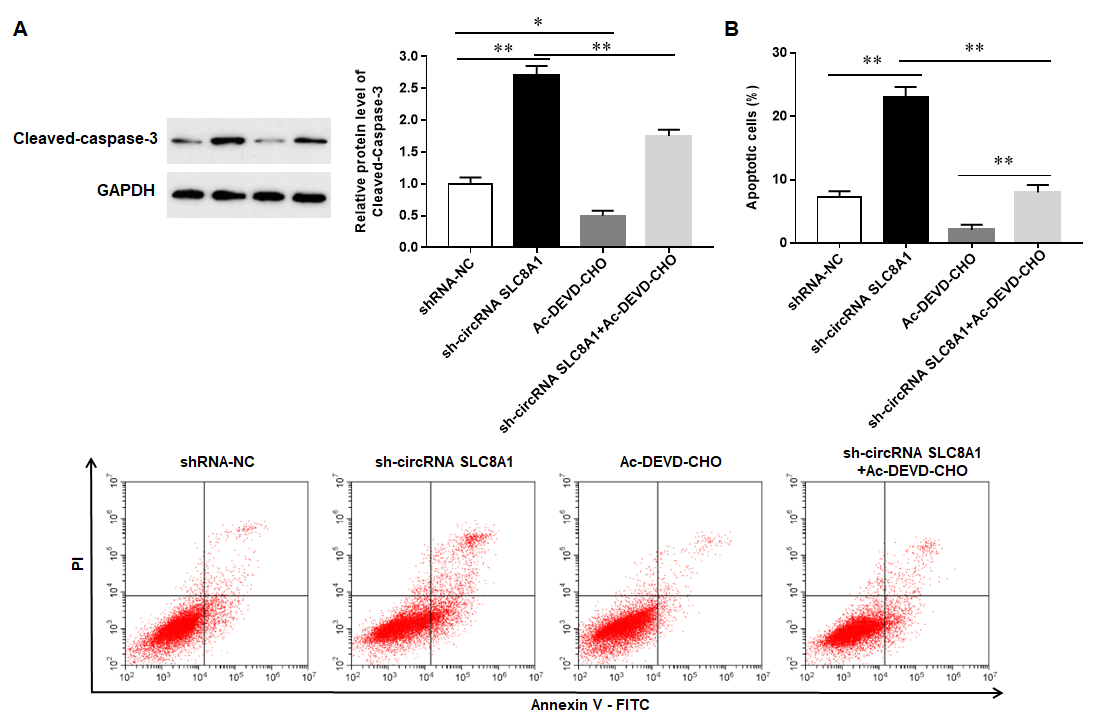


**Supplementary Figure 2. Knock-down of circRNA_SLC8A1 induces the apoptosis of Mtb-infected macrophages by regulating Cleaved-caspase-3 expression.**

The macrophages were transfected with sh-circRNA_SLC8A1 or NC shRNA and treated with or without Ac-DEVD-CHO, a specific caspase-3 inhibitor, and then infected with 5 MOI of Mtb for 12 h. **A**. The expression of Cleaved-caspase-3 protein was detected with Western blotting. **B.** The apoptosis of macrophage**s** was measured with the flow cytometry. Mtb: Mycobacterium tuberculosis. Data were shown as mean ± SEM of one representative experiment, similar results were obtained from three independent experiments. N=5, * *P* < 0.05, ** *P* < 0.01.
